# Tissue distribution of DNA-Hsp65/TDM-loaded PLGA microspheres and uptake by phagocytic cells

**DOI:** 10.1186/1479-0556-5-9

**Published:** 2007-09-20

**Authors:** Ana Paula F Trombone, Celio L Silva, Luciana P Almeida, Rogerio S Rosada, Karla M Lima, Constance Oliver, Maria C Jamur, Arlete AM Coelho-Castelo

**Affiliations:** 1 Departamento de Bioquímica e Imunologia, Faculdade de Medicina de Ribeirão Preto, Universidade de São Paulo, Av. Bandeirantes, 3900, 14049-900, Ribeirão Preto, SP, Brasil; 2 NPT – Núcleo de Pesquisas em Tuberculose – Departamento de Bioquímica e Imunologia, Faculdade de Medicina de Ribeirão Preto, Universidade de São Paulo, Av. Bandeirantes, 3900, 14049-900, Ribeirão Preto, SP, Brasil; 3 Departamento de Biologia Celular e Molecular e Bioagentes Patogênicos, Faculdade de Medicina de Ribeirão Preto, Universidade de São Paulo, Av. Bandeirantes, 3900, 14049-900, Ribeirão Preto, SP, Brasil

## Abstract

This study aimed to demonstrate that microspheres, used as delivery vehicle of DNA-Hsp65/TDM [plasmid DNA encoding heat shock protein 65 (Hsp65) coencapsulated with trehalose dimycolate (TDM) into PLGA microspheres], are widely spread among several organs after intramuscular administration in BALB/c mice. In general, we showed that these particles were phagocytosed by antigen presenting cells, such as macrophages and dendritic cells. Besides, it was demonstrated herein that draining lymph node cells presented a significant increase in the number of cells expressing costimulatory molecules (CD80 and CD86) and MHC class II, and also that the administration of the DNA-Hsp65/TDM and vector/TDM formulations resulted in the up-regulation of CD80, CD86 and MHC class II expression when compared to control formulations (vector/TDM and empty). Regarding the intracellular trafficking we observed that following phagocytosis, the microspheres were not found in the late endosomes and/or lysosomes, until 15 days after internalization, and we suggest that these constructions were hydrolysed in early compartments. Overall, these data expand our knowledge on PLGA [poly (lactic-co- glycolic acid)] microspheres as gene carriers in vaccination strategies, as well as open perspectives for their potential use in clinical practice.

## Background

The encapsulation of DNA into biodegradable microspheres such as those based on poly (lactic-co- glycolic acid) copolymers (PLGA) provides an effective way to protect the DNA against biological degradation by nucleases and permits a continuous release of DNA in a controlled manner over a period of time [1,2]. Besides, these biocompatible and biodegradable copolymers have already been approved for use in humans [3,4]. It has been shown that PLGA microspheres have the potential to act as mediators of DNA transfection targeted to phagocytic cells such as macrophages or dendritic cells [5-7].

Previous studies have demonstrated that mice immunized with microspheres containing plasmid DNA present an immune response to the encoded antigens [8-10]. Our laboratory has focused on intramuscular delivery of the DNA-Hsp65 coencapsulated with trehalose dimycolate (TDM) into PLGA microspheres (DNA-Hsp65/TDM) as a vaccine against tuberculosis [11]. This procedure confers protection against virulent *M. tuberculosis *challenge being particularly effective in inducing the production of high levels of IgG2a subtype antibody and high amounts of IFN-γ. Furthermore, our group has previously shown that this formulation also allowed a ten-fold reduction in the DNA dose when compared to naked DNA-Hsp65 [11].

Although it is clear that encapsulated DNA can stimulate immune response, other aspects of interest including the microspheres distribution and persistence, as well as the kind of cells involved in the uptake process after their *in vivo *administration are poorly understood. PLGA microspheres are thought to be internalized, *in vitro*, by phagocytic cells in a process dependent on their size range (1–10 μm) [12-14], but only few studies were performed *in vivo *to confirm this. The intraperitoneal injection of PLGA microspheres leads to preferential phagocytosis by macrophages, whereas intradermal injection results in uptake of these microspheres by dendritic cells [15]. It has already been demonstrated that the inoculation site also interferes with the biodistribution of DNA-loaded PLGA microparticules [16]. However, the PLGA microspheres biodistribution has not yet been determined. Therefore, a better understanding of the intracellular route and the biodistribution of microspheres may contribute to improving their efficacy and will be the basis for the development of new vaccination strategies. In addition, the study of these important questions in more detail is required to assure the biosafety of microspheres allowing the advance of this technology to a clinical setting against tuberculosis or in veterinary vaccines. In this study we first evaluated the distribution of the PLGA microspheres containing DNA-Hsp65/TDM and then addressed the cells involved in the uptake process following the intramuscular administration. Finally, we have explored the fate of this formulation inside peritoneal macrophages in order to better understand the mechanisms involved in this process.

## Methods

### Plasmid construction

The pcDNA_3_-Hsp65 construct was derived from the pcDNA_3 _vector (Invitrogen, Carlsbad, CA, USA), which was digested with BamH I and Not I (Invitrogen), and a 3.3-kb fragment (corresponding to the *M. leprae *Hsp65 gene) was inserted. The vector pcDNA_3 _was used as a control. DH5α *Escherichia coli*, transformed with either pcDNA_3 _plasmid or with the plasmid containing the Hsp65 gene was cultured in LB medium (Gibco, Grand Island, NY, USA) containing ampicilin (100 μg/mL, Cilinon™). Plasmids were purified using the Endo-Free QIAGEN plasmid purification kit (QIAGEN AG, Basel, Switzerland). Plasmid concentration was determined by spectrophotometry at λ = 260 and 280 nm using the Gene Quant II apparatus (Pharmacia Biotech, Buckinghamshire, UK). The purity of DNA preparations was confirmed by electrophoresis on a 1% agarose gel. Endotoxin levels were determined using a QCL-1000 Limulus amoebocyte lysate kit (Cambrex Company, Walkersville, MD, USA).

### Microspheres preparation

Microspheres were prepared using the double emulsion/solvent evaporation technique. Briefly, 30 ml of dichloromethane solution containing 400 mg of polymer PLGA 50:50 (Resomer RG 505, MW 78 000, from Boehringer Ingelheim, Ingelheim, Germany) and 0.5 mg of TDM (Sigma Aldrich, St Louis, USA) was emulsified with 0.3 ml of an inner aqueous phase containing 5 mg of DNA (pcDNA3 or pcDNA3-Hsp65) using a T25 Ultraturrax homogenizer (IKA, Labortechnik, Germany) to produce a primary water-in-oil emulsion. This emulsion was then mixed with 100 ml of an external aqueous phase containing 3% poly (vinyl alcohol) (Mowiols 40–88, Aldrich Chemicals, Wankee, WI, USA) as surfactant, to form a stable water-in-oil-in-water emulsion. The mixture was stirred for 6 h with a RW20N homogenizer (IKA) for solvent evaporation. Microspheres were collected and washed three times with sterile water, freeze-dried, and stored at 4°C. Fluorescent-labeled microspheres were prepared by adding 6-coumarin (green fluorescence; Sigma Aldrich) to the organic phase. For flow cytometric analysis and microscopy experiments, 6-coumarin/mg polymer was used at doses of 16.5 μg and 0.15 μg, respectively.

Plasmid encapsulation efficiency was determined as described by Barman et al. [17]. Briefly, 2.5 mg of microspheres was resuspended in 0.5 ml of Tris-EDTA buffer, pH 8.0. Chlorophorm, 0.5 ml, was added to the suspension to solubilize the microspheres. The mixture was rotated end-over-end for 60 min at 37°C. The sample was centrifuged at 14000 rpm for 5 min and 0.1 ml of the supernatant was removed for analysis. The amount of DNA (μg DNA extracted/mg polymer) was calculated using the equation: DNA (μg/mg) = (Absorbance_λ = 260 *nm *_× dilution factor)/(εwb); where ε = 50^-1 ^(extinction coefficient of DNA), w is weight of the microspheres in mg, b is optical path length (1 cm).

Particle diameter distribution was evaluated by laser diffractometry in a Cilas 1064 Liquid apparatus (Cilas, France). Results are expressed as the median value of diameter distribution.

### Immunization procedures

Young adult (6 weeks old) BALB/c mice were obtained from the Animal Facilities of the School of Medicine of Ribeirão Preto, University of São Paulo, and were maintained under standard laboratory conditions. Depending on the objective of the experiment one of two protocols was used using three or five animals per group. For biodistribution experiments, mice received a single intramuscular injection of fluorescent microspheres (6-coumarin labeled pcDNA3-Hsp65/TDM) dissolved in 50 μl saline into each quadriceps muscle, whereas the control group received microspheres without the fluorescent marker. For all groups, the amount of microspheres injected was determined based on the encapsulation efficiency rate in order to comprise a 30 μg dose of plasmid (Table 1). At various time points following the administration of microspheres (from day 1 up to 120 days), total cells of several tissues (draining lymph node, spleen, thymus, lung, liver, kidney, testicle and single-cell suspension of bone marrow) were obtained for FACS analysis as described in theprocessing of tissues for biodistribution experiments section. In the second protocol, to determine the leukocyte subsets involved in the uptake process, mice received a single dose of an intramuscular injection of 6-coumarin labeled microspheres containing pcDNA_3_-Hsp65/TDM, pcDNA_3_/TDM (microspheres containing a total dose of 30 μg of plasmid) (Table 1) or empty microspheres (5 mg of the polymer with empty formulation, without plasmid DNA and TDM) (Table 1) in 50 μl saline into each quadriceps muscle. After 7 days, the draining lymph nodes were collected for FACS analysis. Control group was injected with saline. The experiments were in triplicata

**Table 1:** Encapsulation efficiency and median diameter of different microsphere formulation

Microspheres	Encapsulation efficiency	Values of diameter distribution (mean ± SD, μm)
pcDNA3-Hsp65/TDM (6.6 mg 6-coumarin)	49.9% (6.25 μg DNA/mg of microspheres)	3.6 ± 0.27
pcDNA3/TDM (6.6 mg 6-coumarin)	24% (3.0 μg DNA/mg of microspheres)	3.6 ± 0.29
pcDNA3-Hsp65/TDM (60 μg 6-coumarin)	52.8% (6.6 μg DNA/mg of microspheres)	3.9 ± 0.25
pcDNA3-Hsp65/TDM (without 6-coumarin)	34.4% (4.3 μg DNA/mg of microspheres)	3.6 ± 0.22
empty (6.6 mg 6-coumarin)	---	4.8 ± 0.32

### Processing of tissues for biodistribution experiments

At various time points following the administration of microspheres (from day 1 up to 120 days) several tissue types were collected. Before FACS analysis, each tissue received specific treatment in order to collect the total cells. Briefly, the lung was sliced and incubated with 15 ml of RPMI medium plus 1.25 μl of liberase (Roche – Indianapolis IN) for 20 min at 37°C. The sample was washed with RPMI medium supplemented with 10% FCS and was gently sieved to produce a cell suspension. Red blood cells were lysed, the lung cells were treated with Desoxiribonuclease I 0.025% (Sigma-Aldrich – St Louis, USA) and then used for FACS analysis. For liver and testicle, the tissues were sliced, incubated in solution containing 10.8 mg of colagenase IV (Sigma-Aldrich, USA), 22 mg of piruvate (Vetec LTDA, Brazil), 3 mM of calcium chlorate (Sigma, USA) and then submitted to similar procedures as described above for lung. For kidney, the tissue was sliced, incubated in solution containing 10.8 mg of colagenase IV (Sigma-Aldrich, USA), 30 mg of tripsin (BD Difco, USA), 3 mM of calcium chlorate, and then submitted to similar procedures as described above for lung. For draining lymph node, spleen and thymus, the tissues were gently sieved to produce cell suspensions and the red blood cells were lysed. After this, the cells were treated with Desoxiribonuclease I 0,025% (Sigma-Aldrich) and then used for FACS analysis.

### FACS analysis

To asses the biodistribution of the microspheres, an aliquot of 1 × 10^6^cells/tissue from the total cell preparation of the each tissue types was washed three times with PBS and ressuspended in 1% formaldehyde in PBS, and then analyzed by flow cytometry FACScan (Becton Dickinson, San Jose, CA). The green fluorescence signal was analyzed by flow cytometry using Fl-1 channel. For each analysis, 100.000 events were acquired. Analytical flow cytometry was performed using a FACScan (Becton Dickinson, San Jose, CA) and the data were analyzed using the WinMDI software. Results are presented as adjusted percentages, i.e., the background from controls (cells derived from mice that received microspheres without fluorescent marker) was subtracted from the positive percentage. To determine the leukocyte subsets involved in the uptake process, single-cell suspensions (1 × 10^6^cells) isolated from draining lymph nodes were collected and preincubated with anti-CD16/CD32 (Fc Block- 2.4G2 PharMingen, San Diego, CA) during 45 min at 4°C. Then, theses cells were incubated with the specific phycoerythrin (PE) labeled mAbs: anti-CD80, anti-CD86, anti-CD11b, anti-CD11c or anti-CD IAd that was used as phycoerythrin (PE)-conjugate (PharMingen, San Diego, CA) for 30 min at 4°C. The cells were washed with 2% FCS in PBS, resuspended in PBS containing 1% formaldehyde and analyzed by flow cytometry. Analytical flow cytometry was performed using a FACScan (Becton Dickinson, San Jose, CA) and the data were analyzed using the WinMDI software. As described above, the results are presented as adjusted percentages, i.e., the background from the appropriate isotype controls was subtracted from the positive percentage. Statistical determinations of the difference among groups (pcDNA3-Hsp65/TDM, pcDNA3/TDM or empty microspheres) were performed using one-way analysis of variance (ANOVA) followed by Bonferroni post test. All statistical tests were performed with the GraphPad Prism 3.0 software (GraphPad Software Inc). To calculate the mean fluorescence intensity (MFI) increase, the empty microspheres MFI was normalized to 100% and pcDNA3-Hsp65/TDM and pcDNA3/TDM formulations was then expressed as a percentage of empty MFI. The values represented in the graphs are the mean ± SD of three experiments.

### Scanning confocal laser microscopy

Peritoneal macrophages (5 × 10^4 ^cells/wells) were plated, 24 h prior the experiment, on 8 well glass chamber slides (Lab-Tek Chamber Slide System, Nalge Nunc International, Rochester, NY) in RPMI medium, without phenol red, with 10% FCS. Cells were then treated with fluorescent microspheres (pcDNA3-Hsp65/TDM; 1 × 10^5 ^microspheres/wells) at various time points (1, 5, 8 and 10 days), and Texas Red dextran (10.000 MW, 10 μg/mL-Invitrogen, Molecular Probes, Inc., Carlsbad, CA; a marker for late endosomes and lysosomes) was added for the last 24 hours. After this incubation, the cells were washed twice with PBS, fixed for 15 minutes with 2% paraformaldehyde in PBS and rinsed in PBS. Coverslips were mounted using Fluormount (EM Sciences, Hatfield, PA) and examined by confocal microscopy (Leica TCS SP5 AOBS – Leitz, Manheim, Germany).

### Transmission electron microscopy

Peritoneal macrophages (1 × 10^6 ^cells/wells) were plated in 36 mm tissue culture plates, 24 h prior the experiments, in RPMI medium with 10% FCS and treated with fluorescent microspheres (pcDNA3-Hsp65/TDM; 2 × 10^6 ^microspheres/wells) in growth medium at various time points (1, 3, 5, 10 and 15 days). The cells were then washed with PBS and fixed for 2 hours with 2% glutaraldehyde, 2% paraformaldehyde in 0.1 M cacodylate buffer (pH: = 7.4). After this, the cells were post-fixed in 2% osmium tetroxide in 0.1 M cacodylate buffer (pH = 7.4) for 1 h, washed with MilliQ water and stained *en bloc *0.5% uranyl acetate in water.. The samples were dehydrated in a graded series of ethanol solutions (50%, 70%, 90%, 95%, 100%) and infiltrated with propylene oxide. Lastly, the cells were removed from the plates with propylene oxide and embedded in Embed 812 (Electron Microscopy Sciences). Ultrathin sections were cut using an ultramicrotome (Reichert Ultracuts – Leica AG, Germany) and collected on 200 mesh carbon coated copper grids. After staining with uranyl acetate and counterstaining with lead citrate, samples were observed with a transmission electron microscope (Philips 410LS).

## Results

### Characterization of PLGA microspheres

The diameter (1 μm to 10 μm) of different PLGA formulations used in this study presented Gaussian distribution, with mean values ranging from 3.6 μm to 4.8 μm. The DNA encapsulation efficiency ranged from 24% to 52.8% (Table 1).

### Biodistribution of PLGA microspheres after intramuscular delivery

**Figure 1:**
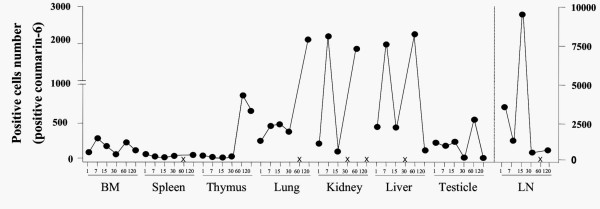
Tissue distribution of PLGA microspheres after intramuscular administration. Mice received a single intramuscular injection of fluorescent microspheres (DNA-Hsp65/TDM). At various time points (between 1 day and 120 days) the total cells from several tissues (draining lymph node, spleen, thymus, lung, liver, kidney, testicle and single-cell suspension of bone marrow) were obtained for FACS analysis (for each analysis, 100.000 events were acquired). Each group consisted of three or five animals. The results were shown as the median of positive values for each time. ×: in this time the results were negative.

FACS analysis was performed in order to investigate the biodistribution of fluorescent PLGA microspheres after intramuscular injection. Lymph node, spleen, thymus, lung, liver, kidney, testicles and single-cell suspension of bone marrow from each animal were harvested at various time points. Our data revealed that the PLGA microspheres presented a widespread distribution, since fluorescent microspheres were detected in all organs analyzed on days 1, 7 and 15 after intramuscular injection (Figure 1). Interestingly, variations in terms of biodistribution were observed among the animals (Table 2 and Figure 1). The microspheres remained in several tissues for extended periods of time. In addition, several organs were still positive to fluorescent microspheres at 30, 60 and 120 after injection, except liver and kidney at day 30, lymph node, spleen and lung at day 60 and kidney at day 120. Lastly, the number of cells containing fluorescent microspheres in the lymph nodes was higher than that observed in other organs (Figure 1).

**Table 2:** Tissue distribution of PLGA microspheres after intramuscular administration

	***1d***	***7d***	***15d***	***30d***	***60d***	***120d***
***L Node***	4	5	2	4	0	2
***B Marrow***	3	1	2	1	2	2
***Spleen***	5	3	2	2	0	1
***Thymus ***	3	2	1	3	4	2
***Lung***	4	5	5	3	0	2
***Kidney***	4	3	3	0	5	0
***Liver***	5	2	3	0	5	2
***Testicles ***	2	4	5	2	5	2

The numbers in the columns indicate the number of the animals that presented positive detection of fluorescent microspheres in each organ evaluated at 1, 7, 15, 30, 60 (n = 5 mice) and 120 (n = 3 mice) days after intramuscular administration.

### Fluorescent PLGA microspheres were captured by antigen presenting cells after intramuscular administration

**Figure 2:**
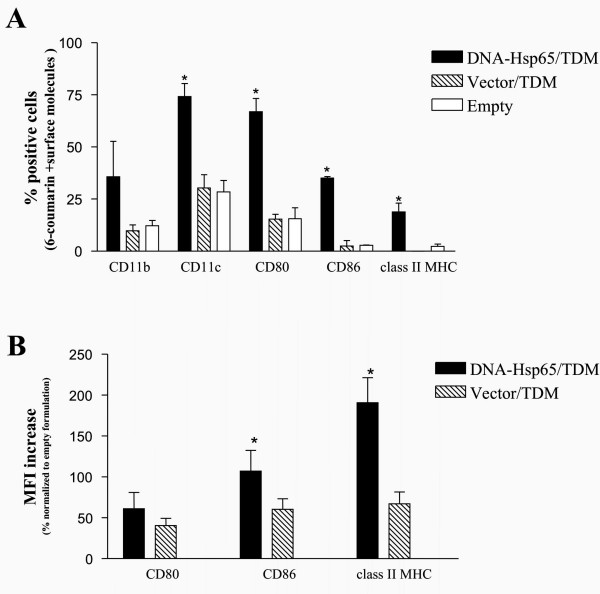
Fluorescent PLGA microspheres were captured by antigen presenting cells after intramuscular administration. To determine the leukocyte subsets involved in the uptake process, mice received a single intramuscular injection of 6-coumarin labeled microspheres containing DNA-Hsp65/TDM, vector/TDM or empty microspheres (without plasmid DNA and TDM). After 7 days, the draining lymph nodes cells were collected for FACS analysis (CD80, CD86, CD11b, CD11c and class II MHC). Control group was injected with saline. (A): Number of cells positive for cell surface molecules CD80, CD86 and MHC class II; *p < 0.05 versus control formulations (vector/DMT and empty). (B): MFI increase expressed as a percentage from pcDNA3-Hsp65/TDM and pcDNA3/TDM formulations, normalized to empty formulation. The values represented in the graphs are the mean ± SD of three experiments.

For FACS analysis, gates (forward and size scatter) were set to eliminate non-ingested particles from the analyzed population. Flow cytometric analysis of the draining lymph node cells revealed that the microspheres were captured by CD11c^+ ^cells and CD11b^+ ^cells, most probably dendritic cells and macrophages (Figure 2A). Moreover, our results demonstrated that draining lymph node cells derived from mice that had received the DNA-Hsp65/TDM-containing formulation presented a significant increase in the number of cells positive for cell surface molecules CD80, CD86 and MHC class II versus the groups that received the control formulations (vector/TDM and empty) (Figure 2A). In addition, the administration of both DNA-Hsp65/TDM and vector/TDM formulations resulted in the up-regulation of the median intensity fluorescence (MIF) of CD80, CD86 and MHC class II expression when compared with empty formulation (Figure 2B). Furthermore, DNA-Hsp65/TDM formulation resulted in a significant increase in the MIF of CD86 and MHC class II when compared with vector/TDM, while CD80 MIF was also found to be increased in Hsp65/TDM group, but this increase was not statistically significant.

### The intracellular compartmentalization of fluorescent PLGA microspheres inside peritoneal macrophage

**Figure 3:**
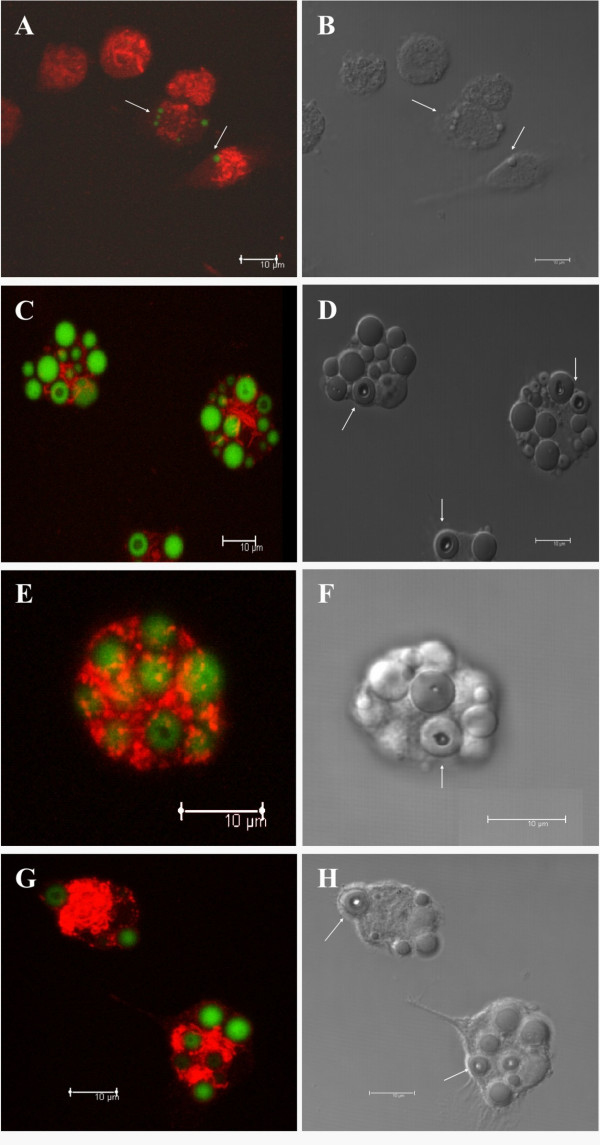
Intracellular compartmentalization of fluorescent PLGA microspheres inside peritoneal macrophages. Confocal images of peritoneal macrophages incubated with fluorescent microspheres (green) for 1 day (A-B), 5 days (C-D), 8 days (E-F) and 10 days (G-H). Texas Red dextran was used as late endossome/lysosomal marker (red). Microspheres did not co-localize with Texas Red dextran in the endo-lysosomal compartment at any of the analyzed time (A, C, E and G). (B, D, F and H): Differential interference contrast microscopy of peritoneal macrophages incubated with microspheres plus Texas Red dextran. Arrows show intracellular hydrolysis of microspheres inside macrophages.

**Figure 4:**
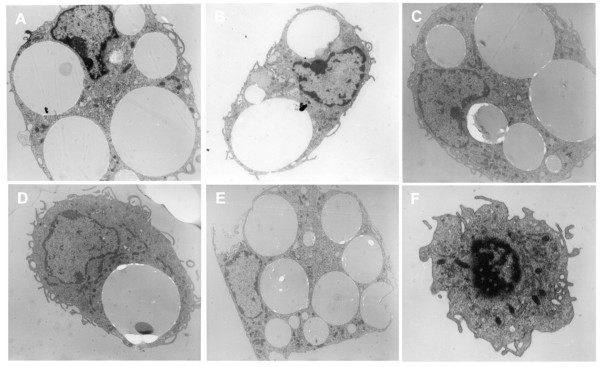
Micrographs of peritoneal macrophages after microspheres phagocytosis. Transmission electron microscopy of peritoneal macrophages incubated with microspheres for 3 days (A), 5 days (B), 8 days (C), 10 days (D) and 15 days (E). Microspheres persisted inside the cells and remained in vesicles during all the time points analyzed. (F): Peritoneal macrophages without exposure to microspheres.

We next investigated the fate of fluorescent PLGA microspheres after their uptake by peritoneal macrophages *in vitro*. Dextran, a marker of late endosomes and lysosomes, was used to localize the microspheres in these compartments. As shown in Figure 3, fluorescent microspheres were not co-localized with Texas Red dextran in the endo-lysosomal compartments at any of the analyzed time (1, 5, 8 and 10 days). In addition, only small microspheres were captured by peritoneal macrophages after 1 day of treatment (Figure 3A–B, arrow), whereas small and large microspheres were captured in other periods of time. It was very interesting to observe intracellular hydrolysis of microspheres inside macrophages after 5 days of incubation (Figure 3D, F and 3H, arrows). These results showing the long lasting persistence of the microspheres inside the cells during all time points analyzed (3, 5, 8, 10 and 15 days – Figure 4) were confirmed by transmission electron microscopy (Figure 4).

## Discussion

DNA-based vaccines have garnered attention for their potential as an alternative treatment for infectious diseases, however, usually multiple doses of high amounts of naked plasmid DNA are required to elicit the desired protective response. Consequently, to optimize this vaccine and reduce the amount of DNA, different methods of delivery, such as gene gun, lipossomes, nano and microparticles, have been used. The encapsulation of the DNA into PLGA microspheres represents an important tool to target phagocytic cells contributing to the development of an appropriate immune response. However, better understanding of the microspheres distribution is necessary to achieve the goal of an effective immunization. With this in mind, we used PLGA microspheres (DNA-Hsp65/TDM), an efficient vaccine against tuberculosis [11], in order to evaluate their distribution, the cells involved in the uptake process and the fate of this formulation inside peritoneal macrophages. For this purpose, PLGA microspheres containing 6-coumarin, a useful fluorescence microscopy probe that is not released from nanoparticles, were used in this study [12].

In the first place, our results demonstrated that soon after intramuscular administration the microspheres were widely spread among several organs, which remained positive to fluorescent microspheres over a follow-up period of 120 days. We hypothesized that this wide distribution occurred via migration of phagocytic cells such as macrophages and dendritic cells, which had captured the microspheres. However, we cannot exclude the possibility that free microspheres might have drained from the injection site into the lymphatics or systemic circulation reaching distant sites where they would be engulfed by phagocytic cells. On top of that, we showed that the number of cells containing fluorescent microspheres in lymph nodes was higher than that observed in other organs. Since lymph nodes are the sites where adaptive immune responses to lymph-antigens are initiated, our findings have far-reaching implications for future research on vaccine design.

Despite the wide dissemination of microspheres throughout the body and their long lasting persistence, previous studies, carried out by our group, demonstrated that the expression of mRNA specific for Hsp65 encapsulated into microspheres (DNA-Hsp65/TDM) was sustained only for 15 days in muscle, liver, draining LN and spleen [11]. After this time, Hsp65 mRNA was not detectable when plasmid DNA encapsulated into microspheres was administrated (data not shown). This fact can be due to the decreased rate of the plasmid DNA delivery at later time points, resulting in lower levels of Hsp65 message that were not detectable in our RT-PCR assay. However, the ability of PLGA microspheres to release the entrapped plasmid DNA slowly indicates that this system is also able to maintain the protein expression for long periods without the need for booster vaccinations.

Similarly to previous observations for microspheres, our group demonstrated that naked DNA (DNA-Hsp65) is detectable in several tissue types, indicating that DNA-Hsp65 is also widely disseminated throughout the body. Notwithstanding, the Hsp65 mRNA was detected up to15 days only in muscle and liver tissues from immunized mice [18].

Herein, we demonstrated for the first time that after intramuscular injection the microspheres were captured by CD11c^+ ^and CD11b^+^antigen presenting cells (APCs) from lymph nodes. Furthermore, the mice that had received the DNA-Hsp65/TDM-containing formulation presented a significant increase in the number of APCs expressing class II MHC and costimulatory molecules (CD80 and CD86) in the lymph nodes. In addition, the expression of class II MHC and CD86 was found to be significantly upregulated (increased MFI) in the APCs from mice that received DNA-Hsp65/TDM. Interestingly, the Vector/TDM formulation also resulted in increased expression of class II MHC and costimulatory molecules when compared to empty formulation, but in a lower extent than DNA-Hsp65/TDM. These results indicate that the upregulation of these molecules was correlated with the presence of Hsp65 protein, which possibly because of a synergistic effect with the CpG motifs present in both the DNA-Hsp65 and Vector constructions. It is possible that after gene transcription the heat shock protein 65 (Hsp65) stimulated phagocytic cells via their cell surface receptors. This fact, which had not yet been described, is in agreement with those reported previously where other Hsps (gp96, Hsp70) are able to upregulate the costimulatory molecules (CD80 and CD86) and MHC class II, probably via Toll-like receptors (TLRs) 2 and 4 [19-21]. These findings prompted the suggestion that TLRs could be involved in the upregulation induced by Hsp65. However, we cannot completely exclude that this upregulation can require the cooperation of other receptors.

Interestingly, after intramuscular immunization a slight variation in the number of microspheres was observed in each organ. This difference may be the consequence of some aspects such as inoculation pressure and/or mouse variation.

The understanding of intracellular trafficking pathways of both DNA vaccine and carrier is of particular relevance. Our *in vitro *results indicate that the microspheres remained in the cells until the last time analyzed (Figure 3 and Figure 4), pointing out a long lasting persistence. Besides, only limited hydrolysis was observed in confocal microscopy (Figure 4). Generally, phagocytozed particles quickly move from early endosomes to late endosomes, and then to the lysosomes. However, we observed that the microspheres were not found in the late endosomes and/or lysosomes, but probably remained retarded in other vesicles, such as early endosomes, until 15 days after internalization suggesting that these constructions were hydrolysed in these vesicles. We propose that similar to other delivery systems involving DNA complexed to synthetic carrier molecules, such as cationic lipids or polymers [22-25], the plasmid DNA was released into the cytoplasmatic compartment. Based on our microscopy data we can infer that the DNA was released into the cytoplasm, whereas the carrier was not. In fact, in spite of evidences of low degree of hydrolysis, which is supposed to allow the DNA escape to cytoplasm, the microspheres remain in cells for prolonged periods of time. It is important to consider that the low rate of microspheres hydrolysis could result in decreased release of DNA into the cytoplasm and therefore result in decreased efficiency of this formulation if theoretically compared with faster release systems. On the other hand, long lasting release of DNA could result in prolonged immune response. Indeed, DNA release strategies are extremely important and must be considered in the rational design of appropriate delivery systems.

Overall, our results demonstrated a wide biodistribution of microspheres, which were preferentially concentrated in the lymph nodes. These findings are of particular importance since the activation of antigen presenting cells can improve the development of specific immune responses independent of the biodistribution. In fact, we not only verified that the microspheres were taken up by macrophages and dendritic cells but we also demonstrated that lymph node cells presented a significant increase in the expression of costimulatory molecules. Likewise, we demonstrated for the first time that after intramuscular administration the DNA-Hsp65/TDM-containing formulation was internalized by antigen presenting cells from lymph nodes. It should be pointed out that the long lasting permanence of the microspheres inside APCs, observed herein, can contribute to the development of an effective immune response without the need of further immunizations. Taken together our results contribute to a better understanding of microspheres as antigen carriers in vaccination strategies and provide additional prospects for their use in clinical practice. The data reported herein will be undoubtedly useful to the design of specific carriers and consequently, to the development of an improved vaccine against tuberculosis.

## Competing interests

The author(s) declare that they have no competing interests.
